# Motivation, Perception, and Behavior of the Adult Orthodontic Patient: A Survey Analysis

**DOI:** 10.1155/2022/2754051

**Published:** 2022-03-04

**Authors:** S. Saccomanno, S. Saran, D. Laganà, R. F. Mastrapasqua, C. Grippaudo

**Affiliations:** ^1^Department of Health, Life and Environmental Science, University of L'Aquila, Piazza Salvatore Tommasi, 67100 L'Aquila, Italy; ^2^Department of Head and Neck and Sensory Organs, Division of Oral Surgery and Implantology, Fondazione Policlinico Universitario A. Gemelli IRCCS, Università Cattolica del Sacro Cuore, Rome, Italy; ^3^ENT Department, Rivoli Hospital, ASL TO 3, Torino, Italy

## Abstract

**Purpose:**

The article is aimed at improving the understanding of the sociocultural profile of adult orthodontic patients and their expectations. In particular, it addresses three main aspects: the motivation and needs that underpin the decision to start orthodontic treatment, how it influences the patients' daily life, and the different oral hygiene demands.

**Materials and Methods:**

An online survey was completed by 276 patients undergoing orthodontic treatment with different techniques. The questions asked concerned gender, age, type of appliance, any previous orthodontic treatments, type of any previous retainers, reasons for therapy, satisfaction, pain, problems in eating, daily number of teeth brushings and flossings before and during the treatment, perception of cost, sensation of visibility of the appliance, and if they would recommend orthodontic treatment.

**Results:**

A significant role within our sample is played by gender; 87.94% consisted of female patients out of which 72.57% wanted to improve their aesthetics, while only 54.84% of male patients cited the same reason. Invisible aligners were preferred by 67.70% of the patients due to them being considered the least painful, causing the fewest problems with eating, and the least visible. Metal braces were perceived as the less expensive treatment. Over a third of the patients (33.85%) had previously undergone orthodontic treatment, among them 54.05% wore a mobile retainer, 31.08% a fixed one, and 14.86% both. Daily tooth brushing and flossing increased during therapy with clear aligners by 48.94% and 126.39%, respectively.

**Conclusions:**

The greatest demand for orthodontic treatments comes from women, as they pay more attention to aesthetics, which makes the clear aligners the most common choice. The relapse after orthodontic treatment seems to cause a higher demand for retreatment, and oral hygiene habits significantly improve during orthodontic treatment, especially with the clear aligners.

## 1. Introduction

The demand for orthodontic treatments by adults, according to the surveys by the American Association of Orthodontists, is constantly increasing: in 2012, US orthodontists had an average of 129 adult patients; in 2014, it was 150; in 2016, it was 173; and in 2018, it was 178, with an overall increase of 27.53% in only six years. Likewise in 1960, the AAO survey reported that only 4.37% of the orthodontic patients were adults, while forty-six years later, according to the 2016 AAO survey, the adult patients were 28.31% of the total with the percentage rising as much as seven times higher.

A study by Muir et al. [1] showed that in 1986, the adult orthodontic patients in New Zealand were 71% female. A similar study was published by Breece and Nieberg [2] which reported that 76% of the adult orthodontic patients of their sample were female and over half were married. The study of Khan and Horrocks [3] confirmed the data of 71% of female adult orthodontic patients. In 2011, Pabari et al. [4] observed a percentage of 73% of females.

The interest of women vs. men in changing their dental look through orthodontic therapies is due to their higher aesthetic standards. Furthermore, a study published by Bolas-Colvee et al. [5] in 2018 revealed that women are more critical of midline diastema, the “black triangle” (the space between gingiva and contact point of upper incisors, when present), and the gingival margin of the upper central incisor compared to men. So, their higher number of requests for treatment can be associated not only to a stronger desire of personal beauty but also to a more acute attention to the objective smile aesthetic details that impact their mouth.

The start of orthodontic therapy represents a novelty that can affect the patient's quality of life [6]. This is related to the visibility of the appliance or braces in the mouth and the fact that improvements are achieved at a slower pace and also that the appliance or braces will be visible for a long time [7, 8].

Oral health influences the physical, social, and psychological well-being. It is undeniable that dental esthetics affect how people are perceived by society and how they perceive themselves [9]. According to Isiekwe et al. [10], there is a strict connection between oral health and quality of life in adults. This study highlights that poor dental esthetics can lead to feelings of shame and it negatively affects the person's image. So, it is clear that dental esthetics influence psychosocial aspects of life, and a good dental appearance can lead to a better social function.

Sun et al. [9] reported that untreated malocclusions are connected to an increased negative impact on the health profile and psychosocial aspects.

Demirovic et al. [11] compared adult patients who received an orthodontic treatment and the ones who did not. The results showed a remarkable negative impact of malocclusion on oral health related to quality of life (OHRQoL) in the ones who did not have therapy and a significant increase of the score on the oral health impact profile (OHIP) [12].

Many adult patients ask for an orthodontic retreatment, due to an orthodontic relapse or unsatisfying previous treatments. Breece and Nieberg [2] in 1986 reported that 6-7% of adult therapies are retreatments. This data was confirmed by Pabari et al. [4] who reported that 12.6% of adult patients declared that they underwent a previous treatment which was not satisfactory.

The possibility to use less noticeable orthodontic solutions rather than the traditional vestibular metal braces persuaded many adult patients to begin orthodontic treatment. The clear aligners are currently the most popular mode, but adult patients appreciate the ceramic and the lingual braces as well. These kinds of appliances work better in adults, as they are more responsible and attentive to the doctor's recommendations in terms of the brackets' care and oral hygiene.

Another issue to be carefully considered in the orthodontic treatment of adults is their discomfort with the appliances. According to Afroz et al. [13], pain can be caused by the presence of the appliances themselves, or by their chairside activation, including enamel interproximal reduction (IPR) procedures.

Proper oral hygiene maintenance during the orthodontic treatment is an important issue for both the clinician and the patients. The choice of esthetic appliances (clear aligners and lingual or ceramic brackets) should be guided by the patient's baseline clinical oral cavity condition which in adults might be worse than in teens. Aljohani and Alsaggaf [14], a cross-sectional study including teen and adult patients, showed a significant improvement in the number of times and duration of daily teeth brushing and in the use of dental floss, so the patient's oral health-related behavior seems to improve during and after orthodontic treatment. In a prospective randomized clinical trial among teen patients, Chhibber et al. [15] found no evidence of differences in oral hygiene levels among patients wearing clear aligners, self-ligated brackets, or conventional elastomeric ligated brackets. However, Albhaisi et al. [16] highlighted the risk of enamel demineralization in people wearing an orthodontic appliance, either fixed or removable, such as a clear aligner.

Having orthodontic therapy may impact eating, as 83.6% of orthodontic patients between 16 and 30 years of age, interviewed by Poudel et al. [17], reported eating difficulties. Albaqami et al. [18] showed an increase of 4.02% in eating difficulties in adolescents with orthodontic appliances than those without. Abed et al. [19] reported that the patients' diet had changed in response to pain and difficulties in biting and chewing, due to the presence of the appliances, and in response to dietary instructions given by the orthodontists.

The purpose of this article is to improve the understanding of the profile of adult orthodontic patients. In particular, this study addresses two main aspects: the motivation and needs that underpin the decision to start orthodontic treatment and how it influences the patient's daily live.

## 2. Material and Methods

An anonymous survey available in four languages (Italian, English, French, and German) was posted into 14 Facebook groups of adult orthodontic patients where they shared their experience about their orthodontic treatment. The patients were asked to complete the questionnaire between January and February 2020, and 257 people answered it from 30 different countries.

All participants provided the informed consent and accepted the privacy policy for the protection of personal data before compiling the survey. No personal information that identifies the individuals was collected, and the data was analyzed only in aggregate form. All responses were collected on an anonymous basis using the Google Form service. The resulting data file that is used for data analysis was free of any identifiers, including mail and IP addresses or other electronic identifiers. The study was conducted in accordance with the principles outlined in the Declaration of Helsinki.

## 3. Consensus

All the orthodontic patients age 18 or older were included in the study, and those surveys lacking an answer to all the compulsory questions were excluded.

The questions asked to the patients were the following:

(1) Are you male or female? (male/female)
(2) How old are you? (18 to 100)
(3) Where are you from? (choice from a list of all the countries of the world)
(4) What orthodontic device do you wear?
  - Metal
  - Ceramic
  - Aligners
  - Lingual
(5) Did you wear orthodontic devices when you were younger? (yes/no)
(6) If you wore braces when you were younger, which retainer did you wear?
  (e) Mobile
  (f) Fixed
  (g) Both
(7) Why have you undergone an orthodontic treatment?
  (h) Occlusion
  (i) Esthetics
  (j) Oral hygiene
  (k) Inclusion
  (l) Prosthetic reasons
(8) Are you pleased with your orthodontic treatment? (0 to 100)
(9) How much pain have you felt during your orthodontic treatment? (0 to 100)
(10) How difficult is eating with an orthodontic device? (0 to 100)
(11) How many times do you brush your teeth daily? (0 to 6 or more)
(12) How many times did you brush your teeth daily before you got an orthodontic device (0 to 6 or more)
(13) How many times do you floss daily? (0 to 6 or more)
(14) How many times did you floss daily before you got an orthodontic appliance (0 to 6 or more)
(15) How expensive do you consider your treatment? (0 to 100)
(16) Would you suggest to your friends that they undergo an orthodontic treatment? (yes/no)

## 4. Statistical Analysis

The normal distribution and equality of variance of the data were preliminarily performed with the Shapiro-Wilk normality test and Levene's test. The one-way analysis of variance (ANOVA) and Scheffe's post hoc comparisons tests were used to evaluate the scores among the groups of subjects receiving different orthodontic appliances, while unpaired Student's test was used to compare scores between males and females after normality tests showed approximate normal distribution. Gender distribution of specific variables was also investigated using chi-square tests.

## 5. Results

We examined the surveys of 257 patients, 31 males and 226 females (M:F ratio 0.13), mean age 33.78 ± 10.7, with a significant difference in age between men (33.36 ± 10.8) and women (25.2 ± 11.38) (Table 1).

Concerning the orthodontic treatments, the majority of subjects (174 or 67.7%) reported wearing clear acrylic aligners, 49 (19.0%) wore metal braces, and 30 (11.6%) wore ceramic braces, while 4 (1.6%) reported wearing lingual braces (Table 1); 87 subjects (33.9%) reported previous treatments when younger (Table 1), and, among them, 40 (54.0%) reportedly used mobile/removable devices and 23 (31.0%) fixed appliances and 11 (14.9%) reported using both (Table 1).

The main reason for choosing orthodontic treatment was esthetics, as reported by 181 subjects (70.4%), followed by occlusion 107 (41.7%), oral hygiene 37 (14.4%), teeth inclusion 33 (12.8%), and prosthetics 7 (2.7%) [Table 1].

Different treatments showed significant differences in mean age, with older patients preferring lingual braces (36.5 ± 12.1) followed by ceramic braces (35.6 ± 11.2), then metal (33.6 ± 11.2), and clear aligner (33.4 ± 10.1) (*p* < 0.05) (Table 1).

Female patients reported higher levels of pain (35.5 ± 20.9 vs. 32.9 ± 27.2, *p* <0.05) as well as eating impairment (52.5 ± 36.1 vs. 39.6 ± 36.1, *p* < 0.05) and social issues (32.7 ± 34.7 vs. 26.1 ± 29.0, *p* < 0.05), while the other scores of the survey showed no significant differences compared to males.

The most expensive treatments reported were lingual braces with a mean score of 85.5 ± 14.4 (rated on a scale of 0-100) followed by ceramic with 80.5 ± 14.1, while aligners had a mean score of 77.3 ± 21.0, and the least expensive treatment reported was with metal braces with a score of 63.0 ± 27.1 (*p* < 0.05) (Table 2).

Patients reported more daily teeth brushing while wearing aligners (3.8 ± 1.1) and lingual braces (3.5 ± 0.5) compared to ceramic (2.9 ± 1.1) or metal braces (2.6 ± 1.0) (*p* < 0.05), while flossing frequency showed no significant differences (Table 2).

The majority of subjects would recommend undergoing orthodontic treatments (242/257, 94.1%), and it was reported more frequently by men (30/31) than women (212/226) (*p* < 0.05).

## 6. Discussion

Research has already highlighted some aspects of the typical adult orthodontic patient [20, 21]. Many have decided to start therapy for esthetic reasons, but 1/3 of the sample indicated they were dissatisfied with the previous treatment. Roughly one-third of the responders reported having previous orthodontic treatments. Possible explanations may be that patients did not use the retainers as they were instructed to do after the end of the treatment, in particular, when the retainers were mobile, as they need significant compliance from the patients. Another reason may be that the final occlusion was not stable enough, thus contributing to a relapse. Furthermore, a physiological factor can determine a relapse, which is the contraction of the bones of both mandible and maxilla with aging [22].

Despite a small portion of the sample reporting wearing lingual braces, patients still reported significant differences in the level of attention towards appearance that can probably affect treatment choices and limit the willingness of using some devices. It appears that lingual braces are characterized by high level of esthetics, but they are also the most expensive, and, even if this is not very relevant, they also have more appeal to people of a higher mean age, even if they make it more difficult to talk and eat compared to the other types. That can be explained by the fact that older adults seek treatments that are the least visible as possible, although the cost is higher than conventional treatments. In fact, the main reason why the patients seek orthodontic treatments is esthetics, and consequently, they are looking for a therapy that impacts their appearance as little as possible while in progress [23, 24]. However, until now and for several reasons, the lingual brackets are not the ones most used, because there are not enough clinicians who have the proper skills to fit them, their cost is quite high, and patients are not familiar with this technique. The most common orthodontic devices remain the metal braces (considered the least attractive), because they have much more clinical history, and almost all orthodontists and patients are more familiar with them.

Another important and relevant element is pain, which seems to be felt more by females than males. This aspect is confirmed by the literature, which highlights that gender does influence pain perception and tolerance. According to Costa et al. [25], the highest level of orthodontic pain was experienced in the first 24 hours after the beginning of the treatment. Their study showed that age and sex were not correlated with orthodontic pain, while there were statistically significant associations with the duration of the treatment and the age of the patient when the appliances were activated. The study by Gao et al. [26] revealed that patients treated with clear aligners experienced lower levels of pain compared to those fitted with fixed appliances.

According to Shaefer et al. [27], there are hormonal factors as well that favor a higher perception of orofacial pain in women than in men. This aspect should be taken into consideration by the clinician in choosing the device and even the pressure applied to the teeth [28].

It is evident from the results of the survey that patients using clear aligners and lingual braces are the ones who are used to brushing their teeth more often, as these devices may require a higher level of oral hygiene for different reasons, as reported by Baron [29]. The patients with aligners can eat without the devices and are usually taught to use them only on a clean mouth, and that determines a higher frequency of brushing. Instead, with the lingual braces, increased brushing time can be attributed to the shape of the device, which can favor the retention of more food particles compared to the vestibular braces [30, 31].

The limit of this research is that the majority of the sample consists of females more than males, even if the literature confirms that the greatest part of the orthodontic patients is composed of females [1].

## 7. Conclusions

This study highlights how important it is for the clinicians to consider the requests of the patients they are treating, because the patients' needs change according to their age, gender, and socioeconomic status [13].

It is remarkable that some kinds of patients are more concerned about the aesthetic aspect of the orthodontic device, likely due to their social and job position, which is something that may warrant further investigation.

Furthermore, it seems very important to adapt the instruments and devices to the kind of patients who are undergoing treatment, because, from this study, it is evident that there are subjects who are more sensitive to pain than others.

Moreover, this study reveals that a significant number of patients underwent a previous treatment. That can be caused by the development of less visible devices, like lingual braces or clear aligners, and by an increasing request for an improvement of the appearance of the smile, which has always been a key factor of social life. The new, less visible devices and the high aesthetic expectation associated with adult awareness may be sufficient motivators for a retreatment.

Finally, the orthodontist needs to keep collaboration alive during the treatment period, and for this purpose, it is very useful to know the reasons that motivate patients to begin an orthodontic therapy or to have a retreatment and the problems perceived by the patients. The ascertained increase of the demand of orthodontic cure is linked to the evolution of different types of the orthodontic tools and devices that can be used nowadays by the clinicians. This study is useful for the management of the orthodontic therapy for the adult patients, who are more conscious and motivated than younger patients.

## Figures and Tables

**Table 1:** Demographic, pain, and treatment data presented as absolute number (percentage).

Demographic	
*Sex*	31 men, 226 women (M : F = 0.13)
*Age*	33.78 ± 10.7
Present treatment	
*Types of treatment*	
Acrylic aligners	174 (67.7%)
Metal braces	49 (19.0%)
Ceramic braces	30 (11.6%)
Lingual treatment	4 (1.6%)
*Motivations of the treatment*	
Esthetics	181 (70.4%)
Malocclusion	107 (41.7%)
Oral hygiene	37 (14.4%)
Teeth inclusion	33 (12.8%)
Prosthetics	7 (2.7%)
Previous treatments	
*Had previous treatment*	87 (33.9%)
*Types of retention*	
Mobile/removable	40 (54.0%)
Fixed	23 (31.0%)
Both	11 (14.9%)
Pain assessment	
*Do or did you suffer from headaches?*	
Before and during with the same intensity	50 (21.5%)
Before and during, but less before	17 (7.2%)
Before and during, but more before	26 (11.0%)
Before the orthodontic treatment	21 (8.9%)
During the orthodontic treatment	35 (14.8%)
Never	87 (36.9%)
*Do or did you suffer from pain during the opening and/or the closure of the mouth?*	
Before and during with the same intensity	13 (5.5%)
Before and during, but less before	8 (3.4%)
Before and during, but more before	9 (3.8%)
Before the orthodontic treatment	15 (6.4%)
During the orthodontic treatment	27 (11.4%)
Never	164 (69.5%)

**Table 2:** Scores of expensiveness perceived and number of times of brushing teeth per day.

	Acrylic	Metal	Ceramic	Lingual	Significance
Mean age	33.4 ± 10.1	33.6 ± 11.2	35.6 ± 11.2	36.5 ± 12.1	*p* < 0.05
Expensiveness score	77.3 ± 21.0	63.0 ± 27.1	80.5 ± 14.1	85.5 ± 14.4	*p* < 0.05
Teeth brushing per day	3.8 + 1.1	2.6 + 1.0	2.9 + 1.1	3.5 + 0.5	*p* < 0.05

## Data Availability

The datasets used and/or analyzed during the current study are available from the corresponding author on reasonable request.
